# Endoscopic submucosal dissection versus endoscopic mucosal resection for the treatment of rectal lesions involving the dentate line

**DOI:** 10.1007/s00464-024-10994-6

**Published:** 2024-06-24

**Authors:** Konstantinos Kouladouros, Johanna Jakobs, Petros Stathopoulos, Georg Kähler, Sebastian Belle, Ulrike Denzer

**Affiliations:** 1 Central Interdisciplinary Endoscopy Department, University Medical Center Mannheim, Medical Faculty Mannheim, University of Heidelberg, Theodor-Kutzer-Ufer 1-3, 68167 Mannheim, Germany; 2 Central Interdisciplinary Endoscopy, Department of Hepatology and Gastroenterology, Charité Universitätsmedizin Berlin, Campus Virchow—Klinikum (CVK), Augustenburger Platz 1, 13353 Berlin, Germany; 3 Endoscopy Unit, Department of Gastroenterology, Endocrinology, Metabolic Diseases and Clinical Infectiology, Marburg University Hospital, Baldingerstrasse, 35043 Marburg, Germany

**Keywords:** Rectal cancer, Rectal adenoma, Laterally spreading tumor, Endoscopic resection, Anorectal junction

## Abstract

**Background:**

The ideal treatment of epithelial neoplastic rectal lesions involving the dentate line is a controversial issue. Piecemeal endoscopic mucosal resection (EMR) is the most commonly used resection technique, but it is associated with high recurrence rates. Endoscopic submucosal dissection (ESD) has been shown to be safe and effective for the treatment of rectal lesions, but evidence is lacking concerning its application close to the dentate line. The aim of our study is to compare ESD and EMR for the treatment of epithelial rectal lesions involving the dentate line.

**Methods:**

We identified all cases of endoscopic resections of rectal lesions involving the dentate line performed in two German high-volume centers between 2010 and 2022. Periinterventional and follow-up data were collected and retrospectively analyzed.

**Results:**

We identified 68 ESDs and 62 EMRs meeting our inclusion criteria. ESD showed a significant advantage in en bloc resection rates (89.7% vs. 9.7%; *P* = 0.001) and complete resection rates (72.1% vs. 9.7%; *P* = 0.001). The overall curative resection rate was similar between both groups (ESD: 92.6%, EMR: 83.9%; *P* = 0.324), whereas in the subgroup of low-risk adenocarcinomas ESD was curative in 100% of the cases vs. 14% in the EMR group (*P* = 0.002). There was one local recurrence after ESD (1,5%) vs. 16 (25.8%) after EMR (*P* < 0.0001), and the EMR patients required an average of three further interventions.

**Conclusion:**

ESD is superior to EMR for the treatment of epithelial rectal lesions involving the dentate line and should be considered the treatment of choice.

Endoscopic resection is the treatment of choice for colorectal adenomas and low-risk early adenocarcinomas [1]. The evolution of local resection techniques has resulted in a wide spectrum of therapeutic options. These include endoscopic mucosal resection (EMR), endoscopic submucosal dissection (ESD), endoscopic full-thickness resection (EFTR), and transanal endoscopic microsurgery (TEM), which alone or in combination with each other and with ablative techniques can effectively treat lesions in the entire colorectum. Nevertheless, some localizations remain challenging even for experienced endoscopists. Epithelial lesions in the lower rectum that are in close proximity to or involve the dentate line have been historically considered difficult to treat endoscopically, mainly because of the poor visualization and maneuverability of the endoscope, resulting in many patients being referred for surgery [2].

Although technical advances in EMR currently yield high success rates of the primary resection, piecemeal EMR (pmEMR) of larger lesions is associated with high recurrence rates [2, 3]. Additionally, the fragmentation of the resected specimen does not allow for an accurate evaluation of the stage and resection status in cases of occult carcinomas, thus leading to patients with potentially endoscopically curable tumors having to undergo oncologic surgery [4]. ESD enables en bloc resection of colorectal lesions regardless of their size, with recurrence rates of 0–2%, and can be curative for low-risk carcinomas. However, as ESD is time consuming and technically challenging, it is not considered first-line treatment in most centers, especially in the Western world [5–7]. A few retrospective studies have demonstrated the advantages of ESD for lesions in the lower rectum involving the dentate line and have shown similar results to procedures performed higher in the rectum [8–10]. However, no studies have directly compared ESD with EMR for the treatment of these challenging lesions. Thus, the aim of our study is to compare the periinterventional and long-term outcomes of ESD and EMR for the resection of epithelial lesions of the lower rectum involving and in close proximity to the dentate line, focusing on technical success, curative resection rates, and local recurrence.

## Materials and methods

### Study design and inclusion criteria

We retrospectively analyzed all patients that underwent endoscopic resection for epithelial lesions located in the lower rectum involving the dentate line at the University Medical Center Mannheim and the Marburg University Hospital between 2010 and 2022. We included all laterally spreading tumors (LST) without macroscopic signs of malignancy with a minimum size of 20 mm and all flat epithelial lesions with suspicion of low-grade malignancy regardless of their size, the anal margin of which was within 2 cm of the dentate line. In all cases the resected area included the dentate line in order to achieve a complete resection of the tumor. The distance of the anal margin of the lesion from the dentate line was measured using the markings on the flexible endoscope. Macroscopic evaluation of malignancy was based on the Japanese Narrow Band Imaging Expert Team—JNET—classification [11].

Approval of the ethics committees of both participating university hospitals was acquired (Ruprecht-Karl University of Heidelberg, file reference 2023-847; Philipps University of Marburg, file reference 23-156 RS).

Based on the type of endoscopic resection, patients were split into two groups: the ESD group and the EMR group. All procedures were performed or directly supervised by four senior endoscopists (K.K., U.D., G.K., S.B.).

### Endoscopic resection technique

#### ESD

**Fig. 1:**
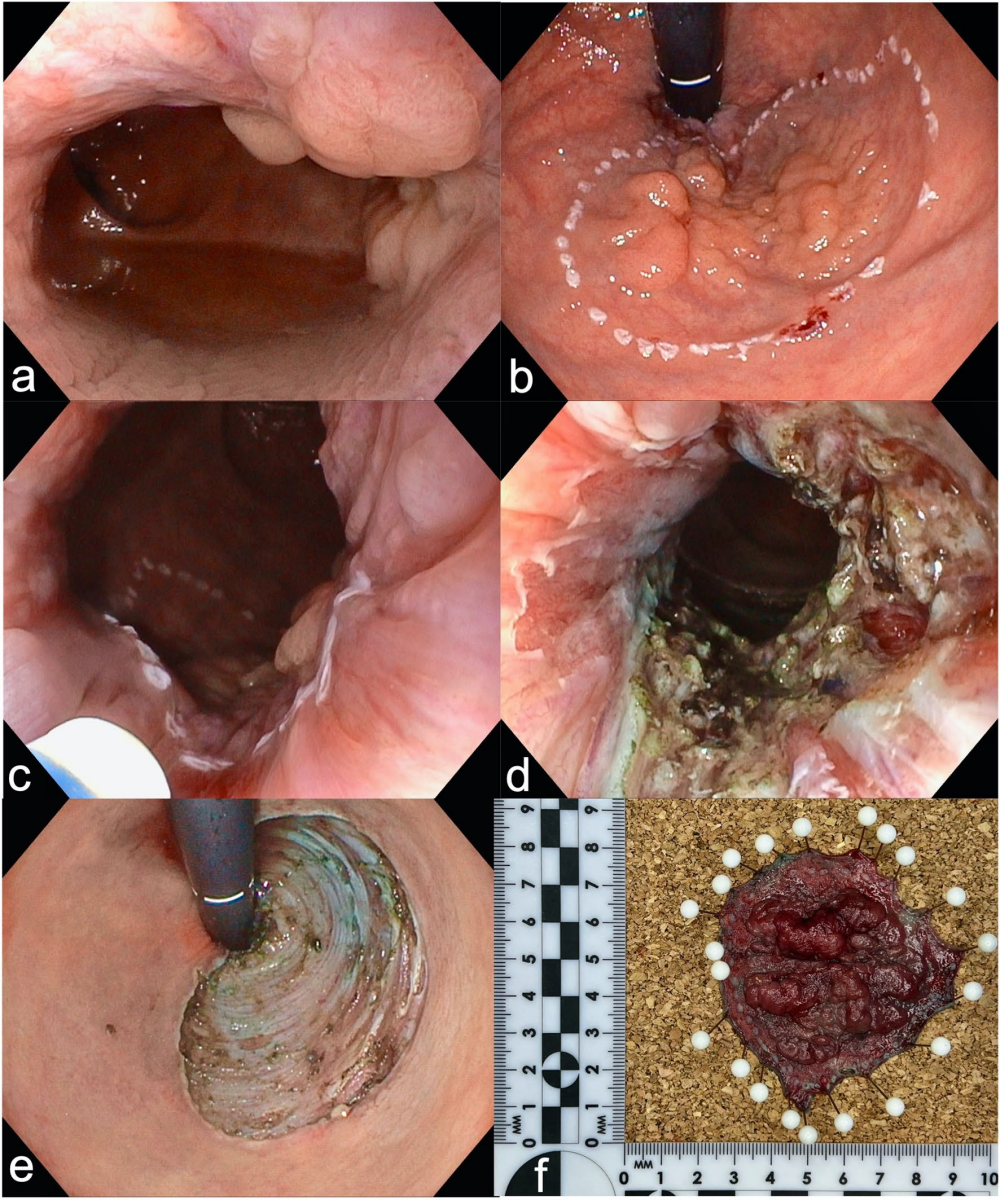
ESD of 45mm large LST granular type, homogenous. **a** The anal margin of the lesion grows into the squamous epithelium of the anal canal. **b** Visualization of the marked lesion in retroflex view. **c** Markings on the anal side of the lesion in the squamous epithelium of the anal canal. **d** Resection defect in the anal canal. **e** Mucosal defect after completion of the ESD in retroflex view. **f** ESD specimen: 45 × 40mm large tubular adenoma with high-grade dysplasia—complete en bloc resection with free deep and lateral margins

**Fig. 2:**
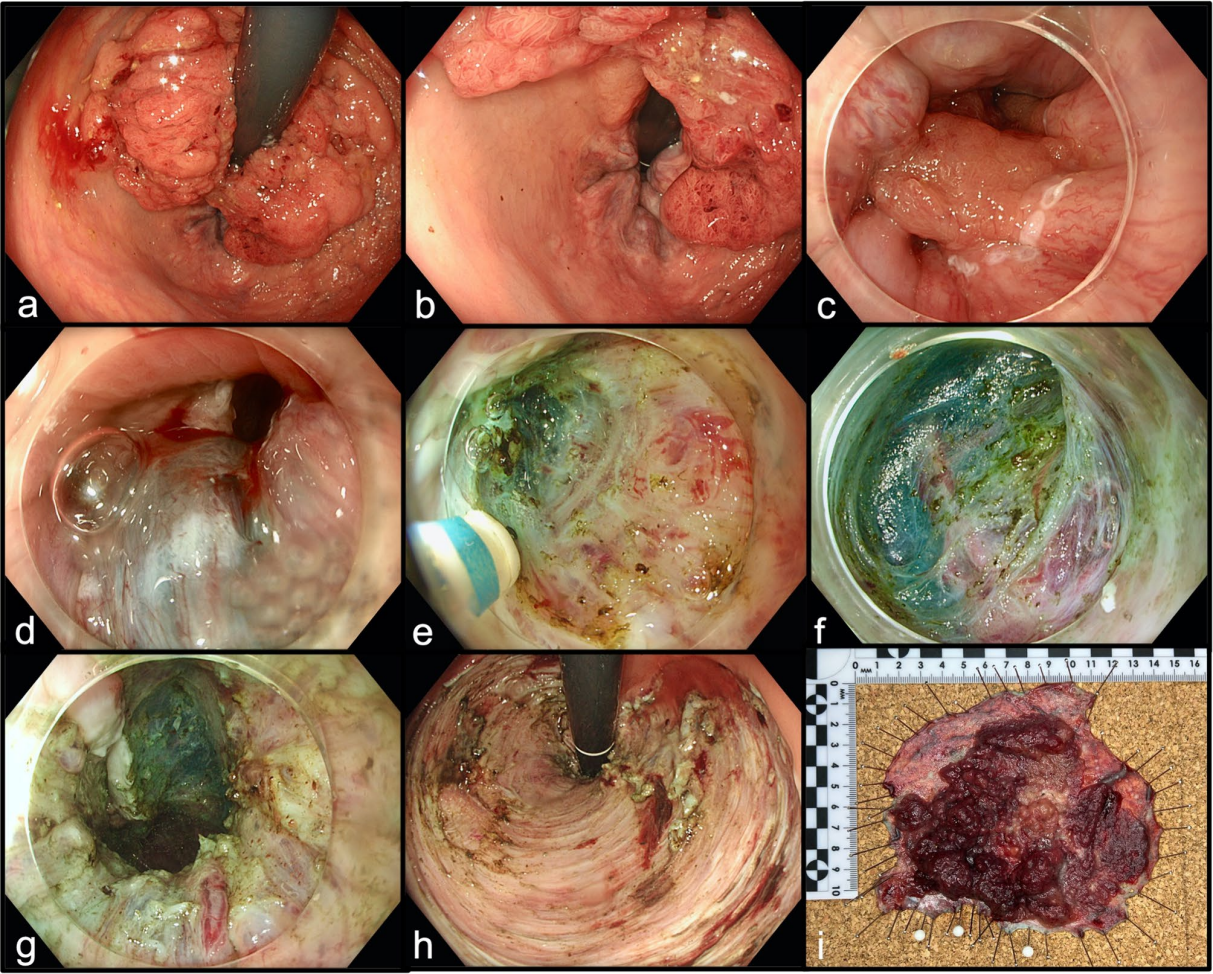
ESD of an 120mm large LST granular mixed type with occult carcinoma (pocket creation method). **a** Visualization of the lesion in retroflex view. **b** The lesion reaches the oral end of the anal canal. **c** Marking of the anal resection margin in the squamous epithelium of the anal canal. **d** Submucosal injection in the anal canal. **e** Epithelial incision and entering the submucosal plane at the oral part of the anal canal. The fibers of the internal anal sphincter are visible. **f** Visualization of the hemorrhoidal veins in the submucosal plane. **g** Epithelial defect in the anal canal. The hemorrhoidal veins are visible. **h** Visualization of the mucosal defect in retroflex view. **i** ESD specimen: a pT1sm1 L0 V0 G2 adenocarcinoma within a 120 × 90mm large tubulovillous adenoma with high-grade dysplasia—complete en bloc resection with free deep and lateral margins (R0)

**Fig. 3:**
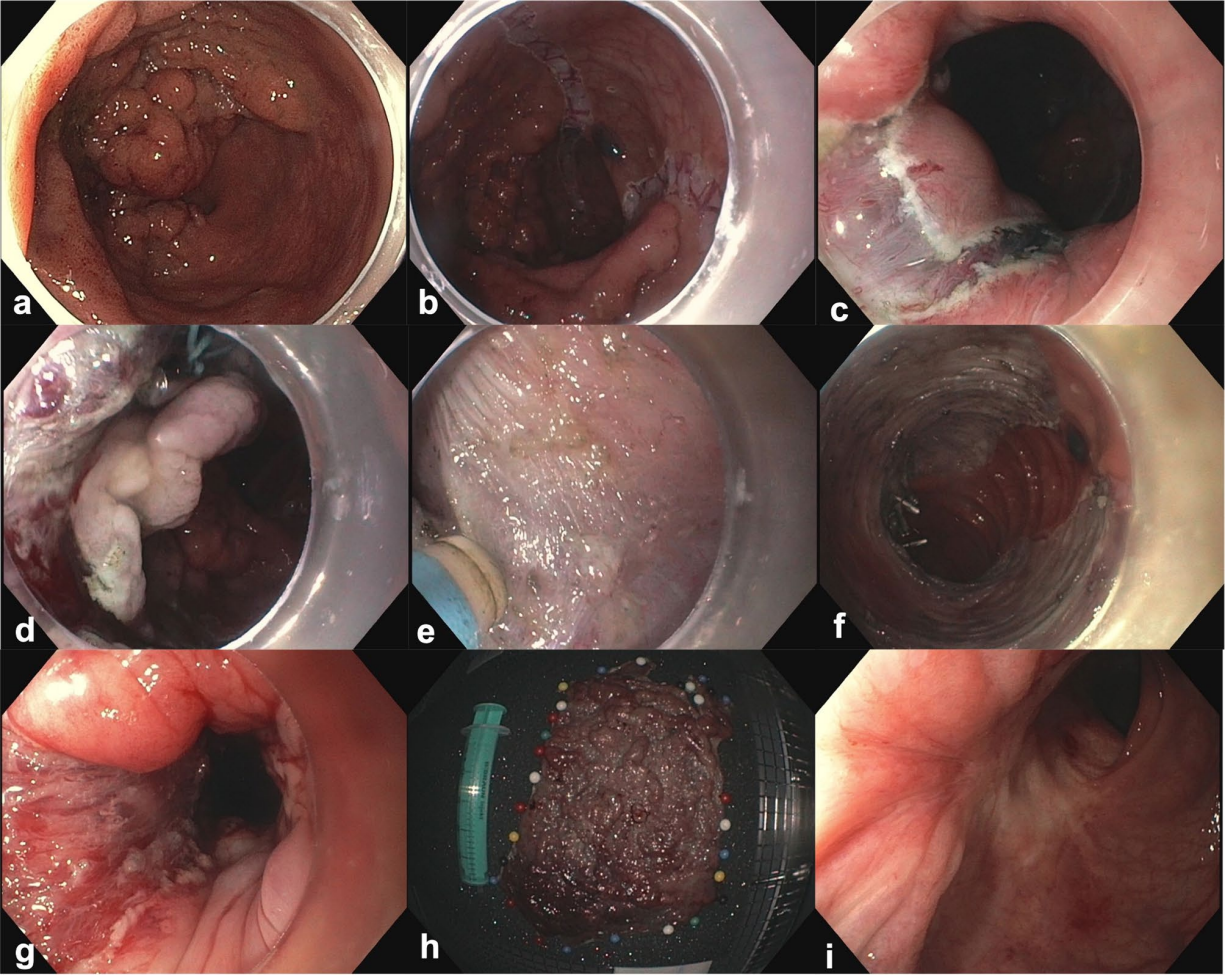
ESD of a 180mm large LST granular mixed type (flap method) with endoscopic follow-up. **a** Visualization of the Lesion in forward view. **b** Circumferential mucosal incision on the oral side of the lesion. **c** Circumferential mucosal incision in the anal canal with visualization of the hemorrhoidal veins. **d** Use of traction (clip-n’-line technique) after initial submucosal dissection in the anal side of the lesion. **e** Exposure of the submucosal plane and the muscle layer with the use of traction. **f** Visualization of the mucosal defect in forward view after prophylactic clipping of large vessels. **g** Visualization of the resection defect in the anal canal. **h** ESD specimen: 184 × 130mm large tubulovillous adenoma with high-grade dysplasia—complete en bloc resection with free deep and lateral margins (R0). **i** Endoscopic follow-up 15 months after the initial procedure showing the scar without any signs of recurrence

Rectal ESDs were performed using a diagnostic or therapeutic gastroscope. The resection margins were marked circumferentially (Fig. 1). Glycerol or plasma expanders with the addition of methylene blue or toluidine blue dye were used for the submucosal injection. Adrenaline 1:100,000 and lidocaine 1% were occasionally added to the solution during submucosal injection in the anal canal. After submucosal injection, the initial, superficial incision was performed using the DualKnife J® (Olympus Europa SE & Co. KG, Hamburg, Germany) starting on the oral side of the lesion, usually in the retroflex position. Circumferential incision was continued to the anal side in the squamous epithelium of the anal canal, with particular caution taken to avoid injury to the hemorrhoidal veins. These were carefully dissected and coagulated before being transected, to avoid excessive intraprocedural bleeding. After the submucosal plane was entered, submucosal dissection was performed until a stable plane was established (Fig. 2). Subsequently, submucosal dissection was continued using the flap or pocket-creation technique, depending on the morphology of the lesion and the endoscopist’s preference. In some cases of flap technique additional traction, usually with the clin-n’-line method, was utilized (Fig. 3). Once the resection was completed, the mucosal defect was carefully inspected and visible vessels were coagulated to prevent secondary bleeding. The resected specimen was stretched, pinned on a cork plate, and photodocumented before being sent for histopathological examination.

#### EMR

**Fig. 4:**
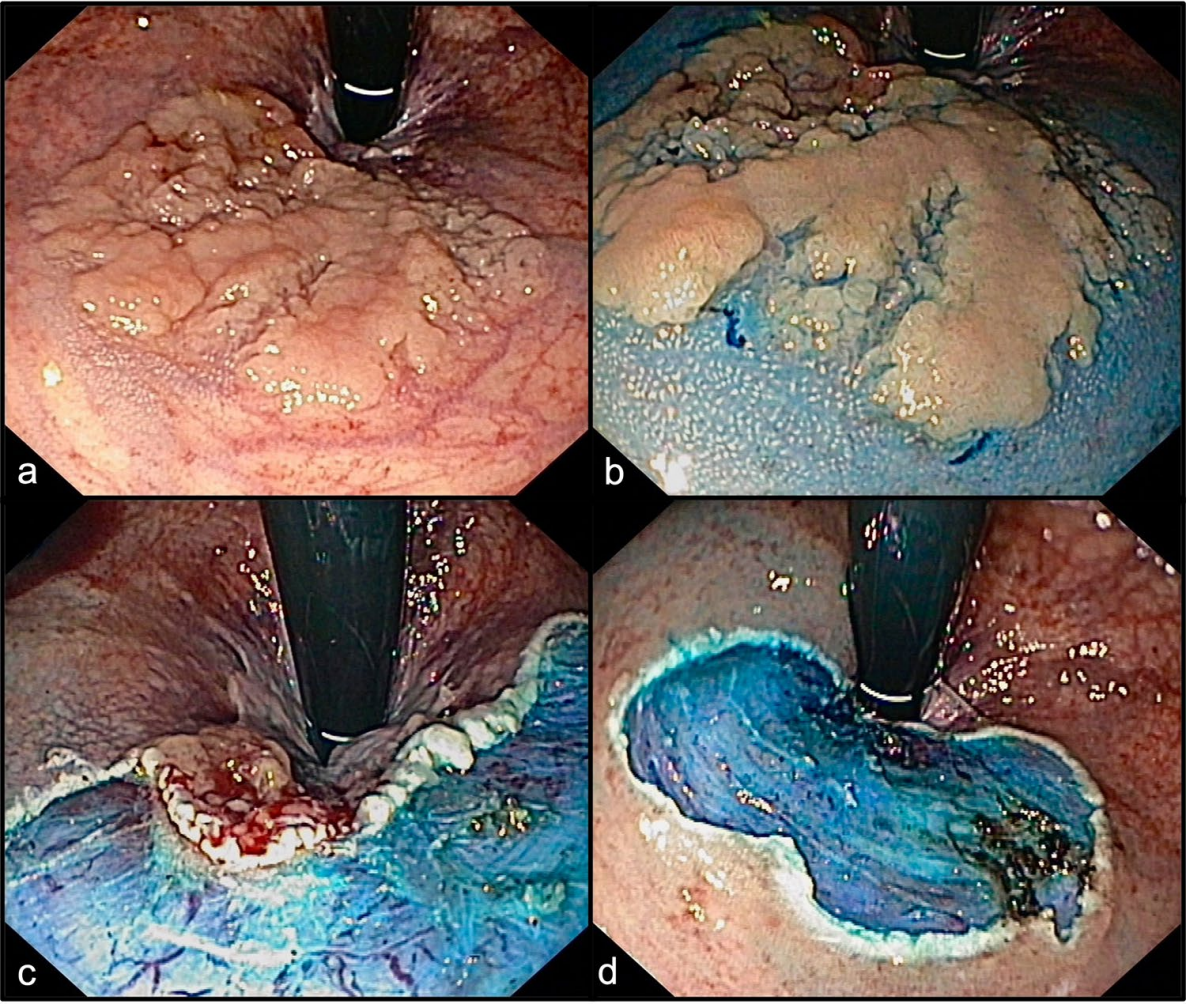
EMR of a 40mm large LST granular type, homogenous (Histology: tubulovillous adenoma with low-grade dysplasia). **a** Visualization of the adenoma in retroflex view. **b** Adequate lifting after submucosal injection. **c** pmEMR beginning on the oral side in retroflex view. **d** Mucosal defect after complete resection of the lesion

A diagnostic or therapeutic gastroscope was also used for rectal EMR. Submucosal injection was performed with either Glycerol/Gelafundin and methylene blue or toluidine blue dye using a standard injection needle or normal saline solution through the HyrdoJet® flexible applicator (Erbe Elektromedizin GmbH, Tübingen, Germany). Adrenaline 1:100,000 and lidocaine 1% were occasionally added to the solution during submucosal injection in the anal canal. Both monofilament and braided snares measuring 15–30 mm were used for the resection, depending on the morphology of the lesion and the endoscopist’s preference. After completion of the resection, the mucosal defect was carefully inspected for remaining tumor tissue and if identified, the tumor tissue was removed with the snare or the biopsy forceps (Fig. 4). After 2018, thermal ablation of the resection margin was performed using the snare tip or argon plasma coagulation. Visible vessels were also coagulated.

### Data acquisition

The periinterventional and follow-up data were extracted from the endoscopic databases and the electronic clinical records of the participating centers (Clinic WinData, E&L GmbH, Erlangen, Germany; ORBIS KIS, Dedalus Healthcare, Bonn, Germany; EPOS, SAP, Weinheim, Germany), anonymized, and retrospectively analyzed.

The following data were recorded for the included patients: basic demographic characteristics; lesion morphology and localization; procedure time; lesion size, histology, and resection status as described in the pathology report; and periprocedural and long-term complications and local recurrence. Follow-up time was defined based on the latest available endoscopic control examination.

The primary endpoints of our study were curative resection rate and local recurrence rate. Curative resection was defined as a macroscopically complete resection with no visible residual tissue for adenomas and as an R0 resection of a low-risk adenocarcinoma. Secondary endpoints included en bloc resection rate, histologically complete resection rate, procedure time, and adverse events.

The collected parameters were compared between the ESD group and the EMR group.

A subgroup analysis was undertaken comparing the curative resection rate between adenomas and carcinomas. In the latter, low-risk carcinomas were defined as well and moderately differentiated (G1-G2) T1 carcinomas with a maximum submucosal infiltration of 2000 µm (T1sm2) without signs of venous or lymphovascular invasion (V0 and L0).

### Statistical analysis

The statistical analysis was performed using IBM SPSS Statistics 28.0. Categorical variables were compared using a chi-square test or Fischer’s exact test, and continuous variables were compared using Student’s t-test or a Mann–Whitney test. Statistical significance was defined as *P* < 0.05.

## Results

We identified 130 patients who fulfilled our inclusion criteria, of whom 68 were in the ESD group and 62 in the EMR group. Age (ESD group: 67 years vs. EMR group: 66 years; *P* = 0.714) and sex distribution (ESD group: 43 male/25 female, EMR group: 33 male/29 female; *P* = 0.247) were similar between the two groups. In the initial study period, between 2010 and 2018, we identified 4 ESD cases and 45 EMR cases, whereas in the later study period, between 2019 and 2020, ESD was more common with 64 ESD cases and 17 EMR cases (*P* < 0.00001). The median size of the resected lesions was similar between both groups (ESD group 36 mm, EMR group 33 mm; *P* = 0.363).

The median distance of the lesions from the dentate line was 1 cm in both groups (*P* = 0.815), and the resection area included the dentate line in all cases. Five lesions in each group were recurrent adenomas after previous pmEMR or TEM (*P* = 0.879). The histological distribution of the resected lesions is depicted in Table 1.

**Table 1:** Histologic distribution of the resected lesions

	ESD (*n* = 68)	EMR (*n* = 62)	*p*
Adenoma	47 (69.1%)	54 (87.1%)	**0.041**
Adenocarcinoma	16 (23.5%)	7 (11.3%)	
Hyperplastic polyp	1 (1.5%)	0	
Anal intraepithelial neoplasia (AIN)	2 (2.9%)	1 (1.6%)	
Squamous cell carcinoma of the anal canal	1 (1.5%)	0	
Neuroendocrine tumor	1 (1.5%)	0	
Among the adenomas:	*n* = 47	*n* = 54	
Low-grade intraepithelial neoplasia	25 (53.2%)	38 (70.4%)	0.075
High-grade intraepithelial neoplasia	22 (46.8%)	16 (29.6%)	
Among the adenocarcinomas:	*n* = 16	*n* = 7	
pTis	6 (37.5%)	2 (28.6%)	0.967
pT1sm1	4 (25%)	2 (28.6%)	
pT1sm2	1 (6.3%)	1 (14.3%)	
pT1sm3	3 (18.8%)	0	
pT1smX	0	1(14.3%)	
pT2	1 (6.3%)	1 (14.3%)	
pT3	1 (6.3%)	0	
G1	1 (6.3%)	0	0.817
G2	14 (87.4%)	7 (100%)	
G3	1 (6.3%)	0	
L0	16 (100%)	7 (100%)	
L1	0	0	
V0	16 (100%)	7 (100%)	
V1	0	0	
Low-risk	11 (68.8%)	6 (85.7%)	0.394
High-risk	5 (31.2%)	1 (14.3%)	

Bold values indicate significance of *p* value

In the ESD group there were nine hybrid procedures, combining ESD with EMR or EFTR. Since our analysis was based on an intention to treat model and hybrid procedures were started as ESD and converted to hybrid, we included these cases in the ESD group. The average procedure time was 145 min in the ESD group and 69 min in the EMR group.

(*P* < 0.0001). A macroscopically complete resection was possible in most cases (ESD group: 67/68, EMR group: 58/62; *P* = 0.140); however, the en bloc resection rate was 89.7% (61/67) in the ESD group in comparison to 9.7% (6/62) in the EMR group (*P* < 0.0001).

The rate of histologically complete resection was significantly higher in the ESD group (72.1%) than in the EMR group (9.7%; *P* < 0.0001). Curative resection rates were similar between both groups considering all tumors (89.7% in the ESD group vs. 83.9% in the EMR group; *P* = 0.324), but ESD showed a significant advantage in the curative resection of adenocarcinomas (62.5% in the ESD group vs. 14.3% in the EMR group; *P* = 0.033). The distribution of resection status and curative resections between the two groups is depicted on Table 2.

**Table 2:** Resection status, curative resections, and adverse events (*Rx: resection status cannot be estimated because of the fractioned specimen)

	ESD (*n* = 68)	EMR (*n* = 62)	*p*
Histologically complete resection	49 (72.1%)	6 (9.7%)	** < 0.0001**
Among adenomas	*n* = 47	*n* = 54	
Free lateral margin	34 (72.3%)	4 (7.4%)	** < 0.0001**
Positive lateral margin	13 (27.7%)	50 (92.6%)	
Among adenocarcinomas (R0)	*n* = 16	*n* = 7	
R0	12 (75%)	1 (14.3%)	**0.032**
R1 (deep margin)	1 (6.25%)	1 (14.3%)	
R1 (lateral margin)	1 (6.25%)	0	
R2	1 (6.25%)	0	
Rx*	1 (6.25%)	5 (71.4%)	
Curative resection	61 (89.7%)	52 (83.9%)	0.324
Among adenomas	47 of 47 (100%)	51 of 54 (94.4%)	0.246
Among adenocarcinomas	10 of 16 (62.5%)	1 of 7 (14.3%)	**0.033**
Among low-risk adenocarcinomas	10 of 11 (90.9%)	1 of 6 (16.7%)	**0.002**
Reason for non-curative resection of adenocarcinomas	*n* = 6	*n* = 6	**0.021**
High-risk histological criteria	5 (83.3%)	1 (16.7%)	
Incomplete resection	1 (16.7%)	5 (83.3%)	
Adverse events			
Delayed bleeding	10 (14,7%)	4 (6,5%)	**0.129**
Perforation	0	0	
Stricture	0	2 (3.2%)	**0.226**

Bold values indicate significance of *p* value

Postinterventional bleeding occurred in 10 cases (14.7%) after ESD and 4 cases (6.5%) after EMR (*P* = 0.129) and could be successfully treated endoscopically, without the need for transfusions or surgical interventions. There were no perforations in either group. Mild anal pain or discomfort was treated with standard analgesics according to the analgetic algorithm of each unit, the use of opioids was not necessary. Two patients in the EMR group presented with a stricture in the lower rectum after EMR, which was able to be successfully treated with endoscopic balloon dilation. There were no strictures in the ESD group (Table 2).

The median follow-up interval was 18 months in the ESD group and 34 months in the EMR group (*P* < 0.0001). There was only one recurrent adenoma in the ESD group vs. 16 cases in the EMR group (25.8%; *P* < 0.0001). There were no recurrent carcinomas. The recurrent adenoma after ESD was identified 12 months after the initial procedure and was treated endoscopically by EMR and avulsion with no further recurrence in the follow-up. The median interval to recurrence after EMR was 6 months (range: 2–18 months) and their further treatment required an average of 3 additional procedures: 11 patients were treated only endoscopically with repeated EMRs and argon plasma ablation, 2 patients were primarily referred for TEM, and 3 patients initially underwent additional EMRs and were finally referred for TEM.

## Discussion

Epithelial lesions in the lower rectum involving the dentate line pose a particular challenge for the endoscopist, and several resection techniques have been suggested for their treatment. We have presented the findings of a bicentral, retrospective study comparing ESD and EMR for the resection of these lesions. To the best of our knowledge, this is the first study to directly compare the two methods. Our data show that ESD can achieve an en bloc resection of the tumors in most cases and is associated with a significantly lower local recurrence rate and a high curative resection rate for locally resectable, low-risk adenocarcinomas, while having a similar safety profile to that of EMR.

The anatomy of the lower rectum and the anal canal is the main reason that interventions in this area are technically difficult. The narrow anal canal with the contracted anal sphincter is followed by the abrupt widening of the rectal ampulla, forming an almost 90° angle. This geometry restricts the view and maneuverability of the endoscope, and some parts of the anorectal junction can only be reached in the retroflex position, which often restricts the function of endoscopic instruments [12]. The presence of the hemorrhoidal veins also increases the risk of intraprocedural and postprocedural bleedings and the complexity of the resection [13]. The direct drainage of these veins to the systemic circulation may also increase the risk of periprocedural bacteremia [3, 8]. Apart from that, the sensory nerve supply of the anal canal is somatic, making procedures in this area painful and thus requiring periprocedural and postprocedural analgesia [3]. Additionally, lesions on the dentate line have been associated with increased risk of submucosal fibrosis, restricting the lifting and visualization of the submucosal plane [14].

EMR is the most commonly used endoscopic resection technique for large colorectal lesions and has been associated with high technical success rates and an extremely favorable safety profile [15]. However, the restricted view and maneuverability of the endoscope in the lower rectum increases the difficulty of this technique even in experienced hands [3]. In a forward view, the almost 90° angle of the rectal wall at the anorectal junction requires an extreme angulation of the endoscope to enable the snare to be pressed against the tissue. The retroflex position offers a better visualization, but the tip of the endoscope is perpendicular to the wall of the lower rectum, which also hampers the positioning of the snare parallel to the lesion. The combination of these factors makes the precise placement of the snare more difficult than in other areas of the colorectum, thus increasing the risk of incomplete resections and injuries of the hemorrhoidal veins. Intraprocedural bleedings from the hemorrhoidal plexus are rarely clinically relevant, but they can quickly reduce the endoscopic view, thus further increasing the risk of incomplete resection. The precision of ESD helps overcome these problems, since the resection margins are carefully marked and followed during the epithelial incision. The reduced overview of the lesion, which is extremely problematic during EMR, is not a restricting factor for ESD because of the close proximity of the endoscope to the tissue required. The initial incision in the anal canal is kept shallow so as not to affect the underlying hemorrhoidal veins, which can then be carefully dissected, coagulated, and transected, thus effectively reducing the risk of intraprocedural bleeding [16]. After the establishment of an initial pocket into the submucosal plane, it is also easier to maintain a stable position of the endoscope and achieve adequate exposure of the submucosal tissue. These technical aspects lead to the increased en bloc and compete resection rates depicted in our data, which are also in accordance with the current literature [12, 17].

Before ESD became popular, TEM was commonly used as an alternative to EMR for the treatment of epithelial lesions in the lower rectum, especially in the suspicion of malignancy. This technique allows for a minimally invasive and precise resection of rectal lesions with the use of a 4cm wide rectoscope and rigid instruments and has been associated with complete resection rates of 83–87% and low recurrence rates [18–21]. Although mucosectomy and hybrid TEM-ESD have been described, most authors advocate for full-thickness resections when performing TEM [22–25]. However, TEM is technically challenging in the lower rectum and especially in the anal canal because of the instability of the rectoscope and must often be combined with transanal excision, which is known to increase the risk of incomplete resections [26–28]. Additionally, full-thickness resection is an overtreatment since a submucosal dissection is enough for the complete resection of all benign and early malignant lesions without deep submucosal invasion. On the contrary, full-thickness resection has been associated with increased scarring in the mesorectum, which can compromise the dissection plane and the outcomes of completion surgery in case of occult high-risk carcinomas [29, 30]. Finally, the insertion of the TEM rectoscope usually requires muscle relaxation and therefore TEM is mostly performed under general anesthesia [26, 31]. The introduction of ESD offered a new method with all the advantages of TEM regarding complete resections and low recurrence rates, while offering better access to difficult localizations like the anal canal, leaving the mesorectum intact and being performed in the endoscopy suite under sedation, thus leading to ESD gradually replacing TEM for most types of lesions in many centers.

The main advantage of ESD, as demonstrated by our findings, is the significantly lower local recurrence rate in comparison to EMR. Although no other study to our knowledge has directly compared these two techniques for lesions involving the dentate line, the single-arm retrospective studies that are available have shown similar recurrence rates to ours for both techniques, thus confirming our findings [2, 3, 9, 17]. As previously mentioned, ESD has an intrinsically higher en bloc resection rate, which is directly associated with a lower recurrence rate, in all parts of the gastrointestinal tract, but this effect is further enhanced in the lower rectum [32]. Additionally, the en bloc resection rate and recurrence rate of ESD are not directly affected by the size of the lesion, as in the case of EMR, thus further highlighting the advantages of ESD for the treatment of large tumors [2, 12, 33]. New developments in EMR, and particularly the addition of thermal ablation of the resection margins, have significantly reduced its recurrence rate, which nevertheless remains at 16% for lesions in the anorectal junction [15]. It must be pointed out that most recurrencies can be treated endoscopically, but this requires the patient’s commitment to an intensive follow-up program in order to detect the recurrence in time [15]. Additionally, the technical challenges of the anorectal junction increase even more in the presence of fibrotic tissue and the absence of lifting usually found in the site of the previous resection. Similar to previous studies, our data show that most of the patients with recurrent adenomas after EMR required multiple further endoscopic procedures and often transanal surgery to effectively treat these lesions, thus increasing the overall treatment costs and reducing patient satisfaction [2].

A subgroup of patients that will particularly benefit from ESD are those with low-risk early rectal cancer, since the resection can be curative and offers a pristine specimen, allowing for the accurate staging and evaluation of the resection status [4, 34, 35]. Epithelial lesions involving the dentate line are reported to have an increased incidence of malignancy with submucosal invasion, which highlights the importance of en bloc and complete resection of those lesions [36]. In our study, almost all low-risk T1 tumors were able to be completely resected, thus sparing the patient a major, amputating surgical procedure with high morbidity and the need for a permanent ostomy. Even in case of a lateral positive margin, careful marking of the specimen can point out the exact area of the resection defect with potential residual tumor, thus offering the possibility of a further endoscopic resection, provided that the histological tumor characteristics are favorable [35]. This is usually not possible after pmEMR, since the quality of the fragmented resection specimen is insufficient for obtaining an adequate estimation of tumor infiltration or resection status [4].

The main arguments raised against ESD are the long procedure times, the technical difficulties, and the high complication rates [3]. Similar to previous reports, our results revealed that ESD in the lower rectum required double the procedure time for lesions of the same size [7, 37]. However, it should be noted that our cohort included the entire ESD learning curve of both endoscopists primarily performing the procedure, and studies have shown that accumulating experience not only improves the outcomes but also significantly reduces procedure time [5, 38, 39]. There is no doubt that ESD procedures in difficult anatomical areas such as the anorectal junction should be performed by experienced endoscopists, but the same applies for EMR, since overcoming the technical challenges described above requires considerable experience [3]. As for periinterventional morbidity, we found no difference between ESD and EMR in our study. The much-feared colon perforation is clinically less relevant in the lower rectum, since it is supported by the mesorectum and peritoneal entry is nearly impossible [7, 9]. The risk of postprocedural bleeding is not negligible, and various studies have shown that it is higher for rectal lesions, particularly those involving the dentate line [9]. However, this is mostly associated with the anatomy of the lower rectum and not with the procedure itself, which explains the similar bleeding rates found in both groups in our study. The same applies for strictures in the anal canal and the lower rectum.

The main strength of our study is the direct comparison of ESD with EMR, with both techniques being performed in two high-volume centers by the same experienced endoscopists, thus contributing to the homogeneity of the groups regarding indication, definitions, and experience of the contributing teams. The multicentric design and the large number of consequent cases included also contribute to the validity of the results. The main limitation of this study is its retrospective nature. The retrospective study design also explains the discrepancy in the median follow-up periods between the two groups, since ESD was primarily performed in the later part of the study period. However, the median follow-up period of the ESD group, although shorter, is still long enough and exceeds the expected interval for recurrence, as demonstrated in the EMR group. Therefore we believe, that the influence of this discrepancy on our findings is minimal. Nevertheless, a prospective, randomized trial comparing these two techniques is required to verify our conclusions.

Based on the findings of our study we believe that ESD is superior to EMR for the treatment of epithelial lesions in the lower rectum that involve the dentate line, especially regarding local recurrence rates and the possibility of curative resection of low-risk early rectal cancer. In experienced hands, ESD is an effective procedure with a safety profile comparable to that of EMR. The long procedure times, the costs involved, and the technical challenges of ESD certainly do not justify its application for all colorectal lesions, and careful patient selection is required in order to maximize its advantages [40]. On the other hand, our data show that patients with lesions located in the anorectal junction are a subgroup who can profit from ESD and therefore we believe that it should be considered as the treatment of choice for those lesions.
